# Better transport accessibility, better health: a health economic impact assessment study for Melbourne, Australia

**DOI:** 10.1186/s12966-019-0853-y

**Published:** 2019-10-22

**Authors:** Vicki Brown, Alison Barr, Jan Scheurer, Anne Magnus, Belen Zapata-Diomedi, Rebecca Bentley

**Affiliations:** 1 Deakin University, Deakin Health Economics, Institute for Health Transformation, School of Health and Social Development, Geelong, Victoria 3220 Australia; 2 Centre for Health Equity, Melbourne School of Population and Global Health, The University of Melbourne, Melbourne, Victoria Australia; 3 RMIT University, Centre for Urban Research, Melbourne, Victoria 3001 Australia; 4 RMIT University, Healthy Liveable Cities Group, Centre for Urban Research, Melbourne, Victoria 3001 Australia

## Abstract

**Background:**

Physical inactivity is a global public health problem, partly due to urbanization and increased use of passive modes of transport such as private motor vehicles. Improving accessibility to public transport could be an effective policy for Governments to promote equity and efficiency within transportation systems, increase population levels of physical activity and reduce the negative externalities of motor vehicle use. Quantitative estimates of the health impacts of improvements to public transport accessibility may be useful for resource allocation and priority-setting, however few studies have been published to inform this decision-making. This paper aims to estimate the physical activity, obesity, injury, health and healthcare cost-saving outcomes of scenario-based improvements to public transport accessibility in Melbourne, Australia.

**Methods:**

Baseline and two hypothetical future scenario estimates of improved public transport accessibility for Melbourne, Australia, were derived using a spatial planning and decision tool designed to simulate accessibility performance (the Spatial Network Analysis for Multimodal Urban Transport Systems (SNAMUTS)). Public transport related physical activity was quantified by strata of age group and sex from Melbourne travel survey data (VISTA survey) and used with the SNAMUTS Composite Index to estimate input data for health impact modelling for the Melbourne population aged 20–74 years. A proportional multi-state, multiple cohort lifetable Markov model quantified the potential health gains and healthcare cost-savings from estimated changes in physical activity, body weight and injuries related to walking to access/egress public transport under two scenarios: (S1) public transport accessibility under current policy directions, and (S2) multi-directional, high-frequency network improvements.

**Results:**

Multi-directional, high-frequency improvements to the public transport network (S2) resulted in significantly greater health and economic gains than current policy directions (S1) in relation to physical activity (mean 6.4 more MET minutes/week), body weight (mean 0.05 kg differential), health-adjusted life years gained (absolute difference of 4878 HALYs gained) and healthcare cost-savings (absolute difference of AUD43M), as compared to business as usual under both scenarios (*n* = 2,832,241 adults, over the lifecourse).

**Conclusions:**

Based on our conservative analyses, improving accessibility to public transport will improve population health by facilitating physical activity and lead to healthcare cost savings compared with business-as-usual. These wider health benefits should be better considered in transport planning and policy decisions.

## Background

Increasing daily physical activity through active transport has been identified by the World Health Organisation (WHO) as a “win-win”, with significant environmental and health co-benefits [1]. Active transport, defined as walking, cycling and using public transport, is associated with health benefits including reduced risk of type 2 diabetes and lower all-cause mortality [2]. Evidence from high-income countries suggests that potential health hazards from a switch from private motor vehicle travel to active modes (e.g. increased risk of transport injury or exposure to pollution) is more than counter-balanced by the health benefits related to increased physical activity [3]. Yet in many countries, including in Australia, transport systems are predominantly motor vehicle-oriented and rates of active transport are low. Of those Australians commuting to work in 2016 79% travelled by private motor vehicle, with only 14% using public transport and 5.2% either walking or cycling [4].

Unlike private motor vehicles, which offer users door-to-door motorised independent mobility, public transport usually requires users to walk for access/egress to shared routes. Public transport use also supports additional walking around and between intermediate destinations during the day, as users are separated from their vehicle at home or point of access. Evidence suggests that public transport users have up to four times greater odds of meeting physical activity recommendations and walk up to 33 min more per day [5, 6] compared to private motor vehicle users.

There is evidence that greater provision of public transport services and better access to stations and stops is associated with higher public transport use [7]. In affluent societies where levels of car ownership are high, good public transport accessibility is a necessary precursor to high levels of public transport ridership, and associated walking. In terms of health outcomes, evidence on the association between public transport accessibility and physical activity is scarcer and less compelling than evidence on the link between public transport use and physical activity, but generally supports the association between increased accessibility and increased physical activity [5, 7, 8]. Improving accessibility to public transport could therefore be an effective policy for Governments to increase population levels of physical activity and reduce negative health externalities of motor vehicle use. Yet to date, limited research has been undertaken on the population health impacts of improvements to public transport accessibility [6, 9].

This paper therefore aims to:

1. derive baseline and two hypothetical future scenario estimates of improved public transport accessibility for Melbourne, Australia;and
2. estimate changes in walking, public transport and motor vehicle travel that foreseeably result from these changes to public transport accessibility; and
3. quantify obesity, physical activity and transport injury-related health benefits and healthcare cost-savings of potential changes in transport behaviours associated with hypothetical improvements to public transport accessibility for the Melbourne, Australia population.

Melbourne is the capital city of the Australian state of Victoria, with a public transport system catering to over 4 million residents [10] and visitors to the city. A radial heavy rail network is complemented by an extensive light rail (tram) system, also largely radial. A patchy bus system of orbital routes connects rail stations and metropolitan activity centres, and provides feeder services to rail. In the past decade Melbourne has experienced the greatest population growth of all Australian capital cities, largely in suburban fringes. Service frequencies in low-density middle and outer suburban regions are generally low and accessibility remains a key issue, with recognised inequities [11]. An inverse care law exists, with those least able to afford private motor vehicle ownership frequently located in outer suburbs, with more affordable housing but low public transport service levels [11].

## Methods

The study utilises two secondary data sets for modelling:

1. A spatial planning and decision tool (SNAMUTS) to characterise baseline and future scenario public transport accessibility indicators for Melbourne, Australia.
2. A household travel survey (Victorian Integrated Survey of Travel and Activity (VISTA)) to provide data on the amount of public transport walking undertaken with different levels of SNAMUTS in the baseline scenario.

The obesity, physical activity and transport injury-related health benefits of hypothetical improvements to public transport accessibility are then estimated for the Melbourne, Australia population.

### Public transport accessibility indicator

The Spatial Network Analysis for Multi-Modal Urban Transport Systems (SNAMUTS) approach uses network analysis methods to measure integral characteristics of public transport systems and their land use context through a series of related accessibility indicators [12]. SNAMUTS offers a planning decision tool based on eight key indicators responding to a range of research policy questions [12]. The SNAMUTS Composite Index is a (weighted) combination of six SNAMUTS indicators: ease of movement (closeness centrality); transfer intensity (degree centrality); land uses within 30-min travel time contours (contour catchment); distribution of travel opportunities over routes and activity nodes (betweenness centrality); the resilience of network elements in the face of future patronage growth (nodal resilience); and the flexibility of users to rely on public transport for travel in any direction (nodal connectivity). All accessibility measures have their limitations, but unlike other much simpler measures of access to public transport used in health research, such as perceived or objective proximity to a stop, SNAMUTS objectively measures the household’s relative accessibility to most destinations across the city by public transport [12]. Hence, it is much better able to capture both the utility of the public transport system in meeting the individual’s travel needs, and how system wide changes to public transport may impact on this accessibility. The decision to use SNAMUTS was also opportunistic: Melbourne is one of a select number of cities across the globe that has SNAMUTS data measuring the accessibility of the current public transport system available, and one of few providing comparable data associated with future transport infrastructure improvements.

SNAMUTS has standard minimum service level inclusion criteria, reflecting the useability of public transport as a full-time regular service suitable for both regular/unplanned and discretionary/unplanned journeys. Mesh Blocks (the smallest spatial units of the Australian census) must have a stop or station: 1) with a benchmark service frequency of at least 20 min in weekday inter-peak and 30 min on weekends for bus/tram, and 30 min for rail; and 2) within walking distance (400 m for bus/tram, 800 m for rail) from the Mesh Block centroid. Mesh Blocks without this were scored as having no minimum service level. The SNAMUTS Composite Index has a maximum score of 60 and was categorised into six brackets: no minimum service, < 10, 10–14, 15–19, 20–24, 25+. These categorisations were most able to capture the variation in accessibility between areas within scenarios, and shifts in accessibility between scenarios, while maintaining sufficient cell size in calculations (Additional file 1).

### Baseline public transport accessibility

Baseline estimates of minutes of daily walking associated with public transport accessibility were generated using data from the 2012–2016 Victorian Integrated Survey of Travel and Activity (VISTA) [13] and the SNAMUTS Composite Index score of the Mesh Block of the VISTA participant’s home address. VISTA is a household travel survey of the travel behaviour of residents in Greater Melbourne and other areas of Victoria, administered by the Victorian Department of Transport [13]. Private households in Mesh Blocks in these areas were randomly sampled using a stratified, clustered design as described elsewhere [13]. As per the methodology of the VISTA survey [13], households were surveyed on their travel and activity patterns for a single designated day. Trip origins and destinations were geo-coded using Geographic Information Systems (ArcGIS 10.2 (ESRI, 2013, Redlands, CA)), and trip distances derived from the shortest street network distance between them, classified by travel mode.

Secondary VISTA data was provided by the Department of Transport, and further cleaned by the researchers. A trip from origin to destination that included any use of train, bus or tram was classified as a public transport trip. Public transport trips using multiple modes were classified as one public transport trip. Of 46,562 VISTA survey respondents, people who did not travel on the survey day (*n* = 10,534) were excluded, as we had no information on their travel patterns, and therefore if they would use public transport with increased accessibility. Children < 18 years (*n* = 7731) were excluded, along with those aged < 20 years or 75 years and over (*n* = 2136) due to small sample size. People living outside of Greater Melbourne (*n* = 1824) were also excluded as they were not covered by the exposure measure. The final sample consisted of 24,337 adults aged 20–74 years living in Greater Melbourne. STATA 14.2 (StataCorp, 2015, College Station, Texas) was used for descriptive and statistical analysis.

### Transport accessibility improvement scenarios

By quantifying the current (baseline) state of accessibility by public transport, as well as the comparative effect of transport infrastructure improvements and land use intensification on the potential accessibility of activity centres, the analysis of future scenarios becomes possible. Scenarios representing hypothetical improvements to public transport accessibility were developed, using information from the Victorian Government’s Rail Network Development Plan [14] and public transport improvements that form part of stated government policy, as well as long-term population and land use projections [15] (Additional file 1).

The first scenario (Scenario 1) represents the trend for public transport accessibility under current policy settings (including the completion of projects that already have funding and implementation commitments) and the patterns of population growth identified under the current trajectories of urban intensification and outer expansion [14, 15].

The second scenario (Scenario 2) represents an aspirational, ‘best-case’ approach, assuming the development of an outer orbital rail link (since announced by state government as a long-term commitment) and a range of inner orbital tram and bus connections to create a more multi-directional, high-frequency network throughout inner and middle Melbourne.

### Methods for estimation of the health-related impacts of the intervention

**Fig. 1:**
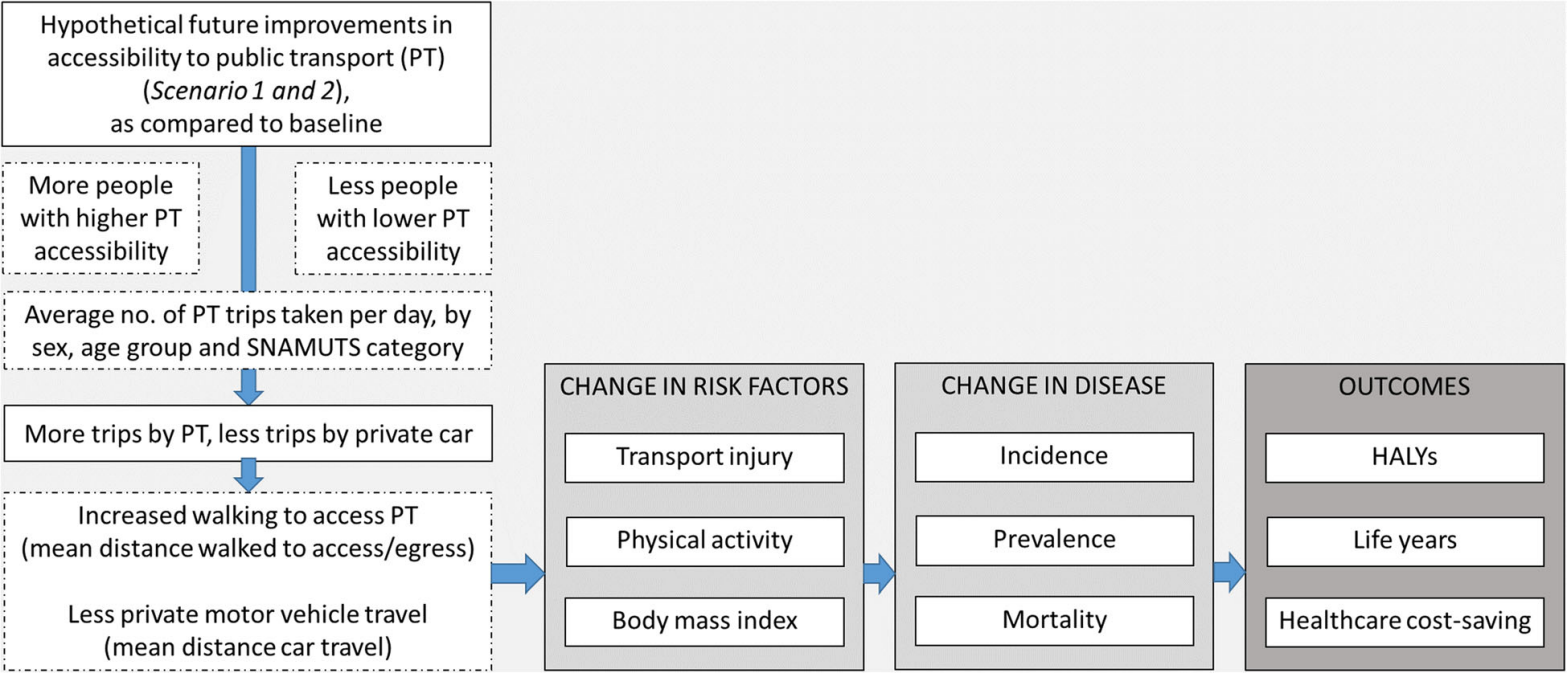
Logic pathway for health impact assessment. HALYs = Health-adjusted life years. PT = public transport. SNAMUTS = Spatial Network Analysis for Multimodal Transport Systems

Figure 1 details the logic pathway used to model the health impacts of hypothetical future improvements to public transport accessibility for the Melbourne population (Scenarios 1, 2). The logic pathway, and the steps followed, are described in more detail below.

#### Prediction of health impacts

The SNAMUTS Composite Index and VISTA travel behaviour data [13] were used to estimate input data for health impact modelling for the Melbourne population aged 20–74 years exposed to the hypothetical accessibility improvements [10] (Additional file 2):

I. Mean baseline number of public transport trips by SNAMUTS Composite Index, by five-year age group and sex;
II. Mean baseline motor vehicle trip distance by SNAMUTS Composite Index, by five-year age group and sex;
III. Mean baseline distance walked to access/egress public transport by SNAMUTS Composite Index, by five-year age group and sex;
IV. Proportion of the sample population in each SNAMUTS Composite Index for the baseline and scenarios 1 and 2, by five-year age group and sex.

Data was extrapolated to be representative of the Melbourne population using the proportion of the sample population in each SNAMUTS Composite Index, by five-year age group and sex and Melbourne population estimates from the Australian Bureau of Statistics [10]. Population estimates were selected for the year 2010, the baseline year for health impact modelling due to the availability of epidemiological data. Therefore the health impacts arising from Scenario 1 or 2 are estimated presuming that the levels of public transport accessibility in Scenario 1 or 2 were applied to the 2010 Melbourne population exposed to the accessibility improvements (*n* = 2,832,241).

The difference in the total number of public transport trips per day between the baseline and each scenario was estimated, by age group, sex and SNAMUTS Composite Index. Estimates of baseline mean distance walked to access/egress public transport were multiplied by the total number of public transport trips taken per day, to estimate the distance walked to access/egress public transport in kilometres per day. The change in distance walked at the population level was calculated per scenario, and distance was apportioned per head of population [10]. We assumed that the modal shift to public transport was in those who had previously travelled by private motor vehicle (Additional file 2).

Physical activity from an increase in walking to access/egress public transport was modelled to a difference in per capita metabolic equivalent task (MET) minutes per week using published values [16, 17](Table 1). The validated energy balance equation by Hall et al. [18] was used to model from a change in physical activity to a change in body weight (kilograms) (Additional file 3). A proportional multi-state lifetable Markov model developed as part of an obesity priority setting study in Australia was used to estimate the obesity, transport injury and physical activity-related health outcomes and healthcare cost-savings over the life course of the population. Details on the model have been published elsewhere [19, 20].

**Table 1:** Key model input parameters

Parameters	Data source and assumptions
Mean number of PT trips per day, by sex, age group and SNAMUTS category	Sampled from a lognormal distribution [13] (Additional file 2)
Mean distance (km), combined PT access/egress by sex and SNAMUTS category	Sampled from a lognormal distribution [13] (Additional file 2)
Mean distance trips by private motor vehicle (km) by sex and SNAMUTS category	Sampled from a lognormal distribution [13] (Additional file 2)
Marginal MET walking from house to car or bus, from car or bus to go places, from car or bus to and from worksite	Sampled from a lognormal distribution, MET value of 2.5 (walking from house to car or bus, from car or bus to and from worksite) with standard deviation 0.75. Sensitivity analysis MET value 4 (walking to work or class), standard deviation 1.6. Values adjusted for inactivity [16], marginal MET 1.5 (sensitivity analysis marginal MET 3).
Total population estimates (population numbers, mortality rates, BMI distribution, PA levels)	Australian Bureau of Statistics [25, 34]
Disease epidemiology, disability weights	Salomon et al. 2012 [35]
Relative risks, total years of life lived with disability	Relative risk uncertainty SE(logRR), Institute for Health Metrics and Evaluation [24], Murray et al. 2013 [36]
Relative risks of PA-related diseases by risk categories	Relative risk uncertainty SE(logRR), Danaei et al. 2009 [37]
Disease healthcare costs	Australian Institute of Health and Welfare [26]
Health Price Index	Australian Institute of Health and Welfare [27]
Transport-related mortality	Australian Road Deaths Database [38]
Transport-related serious injury	Henley et al. 2012 [39]

*Table notes: BMI* body mass index, *Km* kilometres, *MET *metabolic equivalent task, *PA* physical activity, *PT* public transport, *RR* relative risk, *SE* standard error, *SNAMUTS* Spatial Network Analysis for MultiModal Urban Transport Systems

The “relative risk shift” method for the calculation of population impact fractions was used to estimate the consequences of a change in physical activity and body mass index on the incidence of causally related diseases [21]. Diseases causally related to obesity included ischaemic heart disease, hypertensive heart disease, ischaemic stroke, diabetes, colorectal cancer, kidney cancer, breast cancer, endometrial cancer and osteoarthritis. Diseases causally related to low levels of physical activity included ischaemic heart disease, stroke, type 2 diabetes and breast and colon cancer, with an adjustment factor applied to alleviate double-counting of disease-related benefits [22]. Changes in incidence as a result of the hypothetical accessibility improvements result in changes in future prevalence and disease-specific mortality [21].

The ‘risk injury matrix’ approach [23] was adapted to estimate the change in absolute numbers of mode-specific fatalities and serious injuries as a result of the hypothetical shift from private motor vehicle to walking to access public transport. Change in transport injury risk was estimated as the difference between risk from distance travelled by private motor vehicle and distance travelled by pedestrians, based on walking to access/egress public transport. The difference in risk between private motor vehicle travel and public transport was not considered due to the limited availability of mortality and morbidity data on non-road public transport modes (i.e. tram, train). Estimates were incorporated into the proportional multistate lifetable modelling, and compared with baseline mode-specific road traffic accident deaths and years lived with disability obtained from the Global Burden of Disease study [24].

The model used data from the Australian Health Survey 2011–12 [25] and disease epidemiology from the 2010 Global Burden of Disease study [24] (Table 1). Data used to inform the model was national-level, and we assumed that the demographic and epidemiological profile of the Melbourne population was proportionally reflective of that of the Australian population. The comparator was the 2010 Australian population, where the distribution of body mass index, physical activity and road trauma remained unchanged. Data on healthcare costs were obtained for 2001 due to data availability [26], and inflated to 2010 prices using the Health Price Index [27]. Costs and benefits were discounted at 3% [28]. Results are presented as life years gained (i.e. the additional years lived as a result of the hypothetical scenario), health-adjusted life years gained (i.e. the additional years lived adjusted for both the quality and quantity of those additional years) and healthcare cost-savings from diseases prevented, applying to the 2010 Melbourne adult population and reported in 2010 Australian dollars. Modelling was undertaken in Microsoft Excel 2016 and the Excel software add-in Ersatz (version 1.35) was used to undertake Monte Carlo simulation (2000 iterations) to estimate 95% uncertainty intervals around key input parameters. Sensitivity analyses were undertaken assuming postponement of effect (and therefore resultant accrual of health benefits and healthcare cost-savings) for 10 years, to reflect potential timeframes for improvements to public transport infrastructure, and using a less conservative metabolic equivalent task value [16] (Table 1).

## Results

In the baseline data, the mean number of public transport trips taken per day by VISTA participants increased with increasing accessibility, from 0.17 per person in the no-minimum service category to 0.63 per person in the highest category of accessibility (Additional file 2). The mean distance walked for combined public transport access/egress decreased with increasing accessibility, from 1.28 km in areas of no-minimum service to 1.09 km in areas of high public transport accessibility (Composite Index > = 25).

**Fig. 2:**
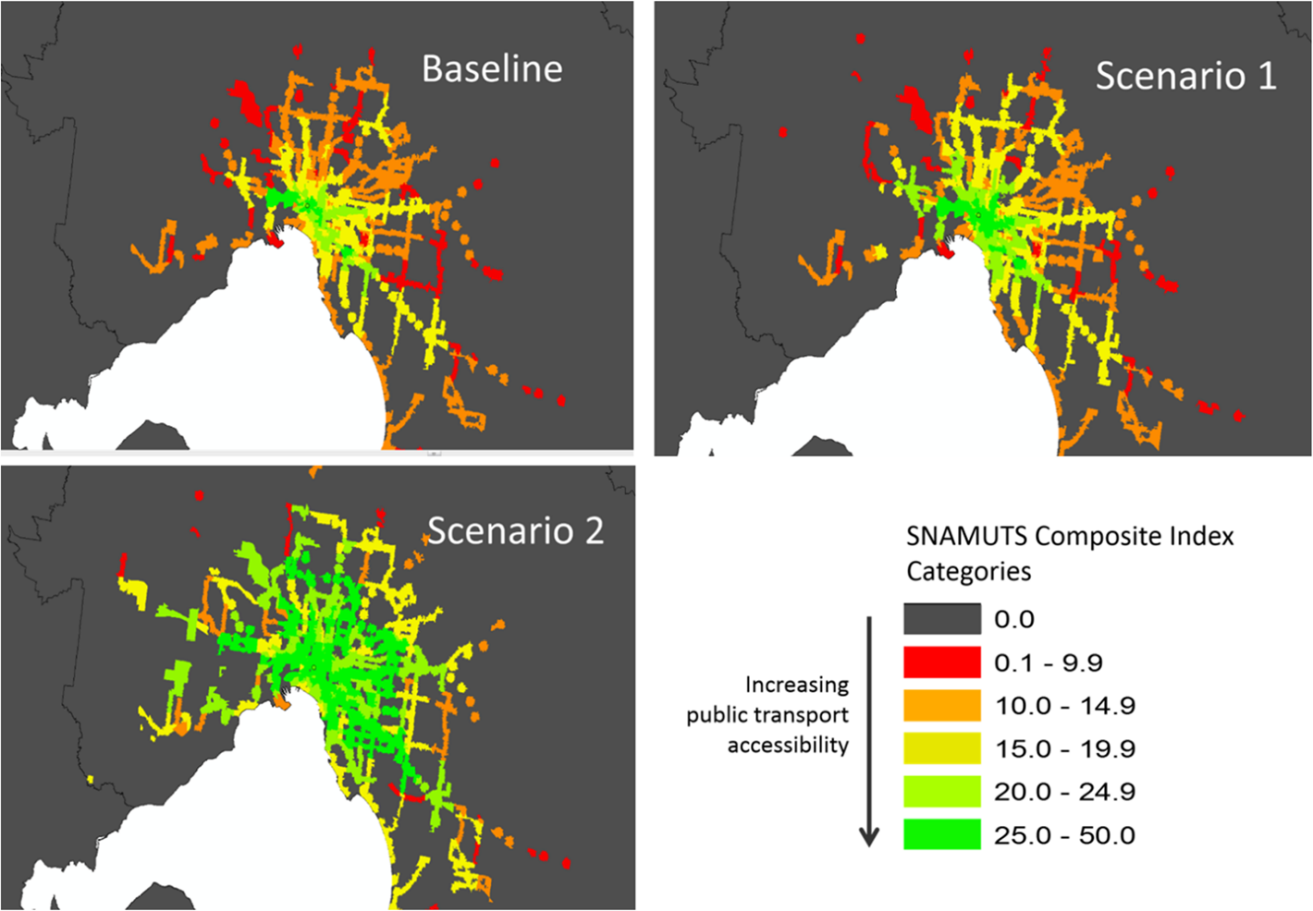
SNAMUTS Composite Index: Baseline Scenario, Scenario 1 and Scenario 2, Metropolitan Melbourne. SNAMUTS = Spatial Network Analysis for Multimodal Transport Systems

There were substantial improvements in accessibility between Baseline and Scenario 2 (see Fig. 2) with the proportion of people in the VISTA sample with the highest public transport accessibility increasing from 2 to 19%. However, many of the gains in accessibility were increases in accessibility from the middle categories of accessibility. Consequently, the proportion of people who did not meet minimum service frequency standards was only reduced from 62 to 54%: over half the sample still did not have minimum service provision in Scenario 2 (Additional file 2).

Under modelled assumptions, improvements to public transport accessibility would lead to a small per capita increase in time spent engaging in physical activity per week (Scenario 1: 1.4 MET minutes/week, Scenario 2: 7.8 MET minutes/week) and a small mean decrease in body weight (Scenario 1: − 0.01 kg, Scenario 2: − 0.06 kg). Scenario 1 population level results are reasonably modest, with 553 health-adjusted life years gained over the lifetime (95% UI 172–1354) and healthcare cost-savings of AUD6M (95% UI AUD2M–15M) (Table 2). Scenario 2 health benefits and healthcare cost-savings are greater, with 5431 health-adjusted life years gained over the lifetime (95% UI 3062-9805) and healthcare cost-savings of AUD49M (95% UI AUD24M–98M).

**Table 2:** Results from health impact modelling over the lifetime (*n* = 2,832,241), Scenario 1 and 2

Results	Scenario 1			Scenario 2		
	Base case	Sensitivity		Base case	Sensitivity	
		Higher MET value	10 years to effect		Higher MET value	10 years to effect
Total life years gained	346 (110–872)	707 (205–1689)	240 (74–584)	4244 (2580-7428)	8545 (5046–15,422)	1451 (436–3592)
Total health-adjusted life years gained	553 (172–1354)	1132 (325–2686)	373 (114–926)	5431 (3062–9805)	10,912 (5990–21,227)	2153 (663–5128)
Total healthcare cost-savings	AUD6M (AUD2M–15 M)	AUD13M (AUD4M–30 M)	AUD4M (AUD1M–10 M)	AUD49M (AUD24M–98 M)	AUD99M (AUD47M–211 M)	AUD23M (AUD8M–56 M)
Difference in transport injury-related mortality* (absolute number)	1 (1–2)	1 (1–2)	1 (1–1)	−4 (− 3 to − 5)	− 4 (− 2 to − 6)	−16 (− 17 to − 15)
Difference in transport injury-related morbidity^a^ (absolute number)	− 195 (− 192 to − 196)	− 193 (− 188 to − 195)	− 199 (− 197 to − 200)	− 1166 (− 1149 to − 1174)	− 1166 (− 1137 to − 1179)	− 1198 (− 1204 to − 1185)

*Table notes:* ^a^ minus means transport injury-related morbidity or mortality savings. AUD: 2010 Australian dollars; *M* million, *MET* metabolic equivalent task. Values are absolute values for the cohort

Results are sensitive to the MET value selected to estimate physical activity effect. Using a higher but still plausible value (Table 1) results in health benefits and healthcare cost-savings that are approximately double that when using a more conservative value (Table 2). Delaying the time to effect of the accessibility improvements for 10 years results in lower estimates for health benefits (Table 2).

## Discussion

Modelled results show small but important health benefits and healthcare cost-savings from relatively conservative improvements to public transport accessibility in Melbourne, Australia (Scenario 1), with larger health benefits and healthcare cost-savings from the development of fully integrated, multimodal public transport networks allowing for significantly improved accessibility (Scenario 2). These results suggest that investment in improving accessibility to destinations across the city by public transport would likely reduce transport-related mortality and morbidity and the burden of diseases associated with physical inactivity and obesity.

This information should be useful for policy-makers, providing quantitative estimates for consideration within the transport planning process. Action point 2.2 of the recent WHO Global Action Plan on Physical Activity 2018–2030 called for improvements to the level of service provided by active transport infrastructure [29]. Yet limited evidence exists quantifying the potential health benefits of improvements to public transport accessibility and associated physical activity [6]. Our analysis demonstrates the complexity of generating this knowledge, given the challenges of collecting rigorous and causative evidence of effect for complex environmental and behavioural changes. Despite these challenges, consideration of the wider health impacts of transport systems and networks is important from a population health perspective, to ensure that transport planning and decision-making encompasses a “health-in-all policies” approach.

Whilst the results presented here suggest the magnitude of potential health-related benefits of policy-relevant improvements to public transport accessibility, they are likely very conservative estimates given that we have included health benefits based on walking only to access/egress public transport. Our results do not therefore account for any indirect walking associated with public transport use, such as walking between intervening places at trip destination as a consequence of separation from private motor vehicles. This is likely to significantly exceed access/egress walking time: in our sample public transport users walked a median 34 min per day, those who did not use public transport walked 4 min per day. If Scenario 2 also resulted in just a half an additional kilometre walked per person per day for indirect travel purposes, the health benefits and healthcare cost-savings of more comprehensive improvements to public transport accessibility would be much higher (62,568 HALYs gained (95% UI 36,739–108,882), AUD576M in healthcare cost-savings (95% UI AUD204M-1B)).

It is also important to note that our modelled estimates do not capture some of the other potential benefits of improving accessibility to public transport. For instance, we do not include any potential impacts on productivity that may arise from reductions in obesity and road trauma and improvements in physical activity. Our estimates also do not account for potential benefits such as decreased travel time and congestion or improvements to air quality, arising from a shift from private motor vehicle transport to more public transport use.

Our study was limited by its hypothetical nature, and the use of population level modelled effect sizes to estimate health benefits and healthcare cost-savings. Due to the hypothetical nature of the modelled scenarios causative, “gold standard” evidence of effect is not available. Our modelled population level effect sizes are based on transport planning projections, the best available evidence on existing transport behaviours and logic pathways to potential physiological effects. Given the high level of uncertainty of effect of these hypothetical improvements, it is therefore important that results be cautiously interpreted. While we used the best available data to estimate the healthcare cost-savings of diseases averted, we acknowledge that this data source is from 2001 and healthcare costs may have changed [26]. We adjusted these prices to the reference year using appropriate methods, but this limitation should also be taken into account when interpreting results.

Notably, Melbourne is a low density and sprawled city and even with the extensive changes to the public transport system under Scenario 2, more than half of participants did not meet minimum service standards. The SNAMUTS tool captures those components of a public transport system that offer regular, full-time services that enable users to rely on public transport for both regular and discretionary journeys. In many low-density areas, particularly in outer Melbourne, typically hourly bus services with limited or no presence on evenings or weekends fail to meet this standard. The orbital public transport links in Scenario 2 address this shortfall across Melbourne’s inner to middle suburbs, but rolling out similar improvements across all outer and peri-urban suburbs would require a level of additional operational resources (and thus public subsidies) that decision makers currently do not appear to be prepared to provide. Our analysis assumes that accessibility (or lack thereof) to public transport is a significant deterrent to usage for people with low baseline accessibility, and that improved accessibility will yield public transport patronage rates similar to those with high baseline accessibility. We also assume that improved accessibility in outer areas of Melbourne will result in similar travel patterns and behaviours as in those areas already better-served by public transport, and would not equate to adapted behaviours in outer areas that may reduce the potential for increased physical activity (for example, higher usage of “park-n-ride” facilities). Clearly more evidence is required from longitudinal studies, capitalising on natural experiments and using rigorous methodologies to collect and analyse comprehensive data on the full range of potential impacts. Finally, the use of 2010 as the baseline year for health impact modelling implies that little improvement has been made to public transport systems in Melbourne between 2010 and now, although evidence suggests that marginal improvements have been made [30].

The review by Sener at al. [6] found limited research related to the health benefits of public transport use, let alone improved accessibility to public transport. Several studies have estimated the potential health benefits and healthcare cost-savings arising from hypothetical shifts from private motor vehicle travel to active transport in Melbourne [31, 32] or other Australian cities [33], but not resulting from improvements to public transport accessibility. Brown et al. [32] applied a published estimate of body mass index effect to a hypothetical 5% increase in the Melbourne working age population using active transport and estimated lifetime health benefits of approximately 1602 health-adjusted life years gained (95% UI 1165–2086) and healthcare cost-savings of AUD19M (95% UI AUD14M–24M, AUD2010 prices). Zapata-Diomedi et al. [33] estimated 32,600 health-adjusted life years gained (95% UI 16,600–46,800) and healthcare cost-savings of AUD312M (95% UI AUD173M-463M,) if government targets for reduction in private motor vehicle travel and increases in walking, cycling and using public transport were achieved in Brisbane, Australia (*n* = 860,000 adults exposed to the intervention). The results presented here are not generalizable to other Australian cities due to the contextual nature of the modelling. However, with some work the methodology employed (i.e. use of the SNAMUTS Composite Index and hypothetical scenario modelling based on best available data to estimate obesity, physical activity and injury-related health benefits and healthcare cost-savings) is replicable both domestically within Australia and internationally.

## Conclusions

With very little shift in the proportion of Australians meeting their physical activity guidelines despite more than 20 years of public health interventions, opportunities for increasing physical activity through active and public transport should be an important focus for public health researchers and policy makers. Our results give a conservative indication of the wider health-related benefits of hypothetical but policy-relevant improvements to accessibility to public transport for the Melbourne population. Results suggest that the greatest share of health benefits and healthcare cost-savings arise from comprehensive transport systems that encourage active transport as a habitual, sustainable, convenient and affordable mode of transport for daily living. Modelled results provide valuable information for decision-makers, and perhaps more importantly, demonstrate the benefits and limitations of quantifying the impact of transport on people’s health.

## Supplementary information

**Additional file 1.** Modelling of changes to the public transport system in Melbourne and development of the SNAMUTS scenarios. Describes the SNAMUTS scenarios in further detail. (DOCX 15 kb)

**Additional file 2.** Input data used in the estimation of effect for health impact modelling. Lists input data for estimation of health effects. (DOCX 16 kb)

**Additional file 3.** Estimation of physical activity and obesity effect. Provides estimates of physical activity and obesity effect. (DOCX 17 kb)

## Data Availability

The datasets used and/or analysed during the current study are available from the corresponding author on reasonable request. The model is also available from the corresponding author on reasonable request.

## Abbreviations

AUD: Australian dollars
B: Billion
HALYs: Health-adjusted life years
M: Million
MET: Metabolic equivalent task
SNAMUTS: Spatial Network Analysis for Multimodal Urban Transport Systems
VISTA: Victorian Integrated Survey of Travel and Activity
WHO: World Health Organisation
