# Supplementary material for: Better transport accessibility, better health: a health economic impact assessment study for Melbourne, Australia
Source: Int J Behav Nutr Phys Act. 2019 Oct 22;16:89. doi: 10.1186/s12966-019-0853-y (PMC6805526; doi:10.1186/s12966-019-0853-y)
Supplement: Supplementary file 1 — Additional file 1. Modelling of changes to the public transport system in Melbourne and development of the SNAMUTS scenarios. Describes the SNAMUTS scenarios in further detail. (DOCX 15 kb) [file 12966_2019_853_MOESM1_ESM.docx]

**Additional file 1: Modelling of changes to the public transport system in Melbourne and development of the SNAMUTS scenarios**

The SNAMUTS scenarios for calculating public transport accessibility levels across Melbourne have been derived as follows:

The *Baseline Scenario* describes the actual network configuration and service levels in 2016, as per published Public Transport Victoria (PTV) timetables and network maps. This data is paired with residential data (per Mesh Block) and employment data (per Destination Zone) from the 2016 Australian Bureau of Statistics census (1).

*Scenario 1* describes the projected network configuration and service levels in 2026 in line with committed infrastructure projects and strategies as per current government policy. These projects include the Melbourne Metro Tunnel currently under construction and slated for completion in 2025, alongside a suite of rail network reconfigurations and service improvements identified in Stage 2 of the PTV Rail Network Development Plan (2). Changes to the tram and bus network and service levels have been modelled to reflect the medium-term intentions of the tram operator (Yarra Trams) and the State Government planning layer (3) for a minimum service standard along the Principal Public Transport Network (PPTN). Residential and employment figures have been extrapolated from the 2016 census by using growth rates for local areas in 2026 as specified in the Victoria in Future project (4).

*Scenario 2* includes all measures from Scenario 1 and a suite of further aspirational infrastructure projects and service improvements beyond 2026 derived from long-range government policy and public transport advocacy. These are primarily derived from the network and service measures in the final Stage 4 of the PTV Rail Network Development Plan (5), and a package of additional inner urban orbital tram and bus extensions and service improvements detailed in Scheurer et al (6). Residential and employment projections are arrived at by the same method as in Scenario 1, but using growth rates for 2036 from the Victoria in Future project (4).

**REFERENCES**

1. Australian Bureau of Statistics. 2016 Census Canberra, Australia: ABS; 2017 [Available from: <http://www.abs.gov.au/websitedbs/censushome.nsf/home/2016>.

2. Public Transport Victoria. Network Development Plan Melbourne, Australia: PTV; 2012 [Available from: <http://www.ptv.vic.gov.au/about-ptv/data-and-reports/growing-our-rail-network-2018-2025/>.

3. Department of Transport. Principal Public Transport Network 2018 [Available from: <https://transport.vic.gov.au/about/planning/principal-public-transport-network>.

4. Department of Environment Land Water and Planning. Victoria in Future 2016. Melbourne: DELWP; 2017.

5. Public Transport Victoria. Rail Network Development Plan. Melbourne: PTV; 2012.

6. Schurer J, Curtis C, McLeod S. Making Melbourne's public transport network multi-directional: Can the associated accessibility boost mobilise latent potential for ridership and city-building? 38th Australasian Transport Research Forum (ATRF); Melbourne2016.
