# Supplementary material for: Better transport accessibility, better health: a health economic impact assessment study for Melbourne, Australia
Source: Int J Behav Nutr Phys Act. 2019 Oct 22;16:89. doi: 10.1186/s12966-019-0853-y (PMC6805526; doi:10.1186/s12966-019-0853-y)
Supplement: Supplementary file 2 — Additional file 2. Input data used in the estimation of effect for health impact modelling. Lists input data for estimation of health effects. (DOCX 16 kb) [file 12966_2019_853_MOESM2_ESM.docx]

**Additional file 2 -** **input data used in the estimation of effect for health impact modelling**

Table 2.1: Results of input data for estimation of effect for health impact modelling

| **Sex** | **Variable** | **SNAMUTS category** | | | | | |
| --- | --- | --- | --- | --- | --- | --- | --- |
|  |  | **No minimum service** | **Comp<10** | **Comp 10-14** | **Comp 15-19** | **Comp 20-24** | **Comp 25+** |
| **Males** | **Proportion of population, baseline** | 62% | 4% | 15% | 13% | 4% | 2% |
|  | **Proportion of population, Scenario 1** | 61% | 3% | 13% | 13% | 7% | 3% |
|  | **Proportion of population, Scenario 2** | 54% | 1% | 4% | 10% | 12% | 19% |
|  | **Mean number PT trips taken per day**  **(95% UI)** | 0.18  (0.17-0.19) | 0.18  (0.13-0.23) | 0.29  (0.26-0.33) | 0.35  (0.32-0.40) | 0.52  (0.44-0.61) | 0.60  (0.47-0.76) |
|  | **Mean distance walked (km), for combined PT access/egress (95% UI)** | 1.36  (1.28-1.44) | 1.12  (0.96-1.31) | 1.12  (1.04-1.20) | 1.18  (1.11-1.25) | 1.11  (1.01-1.22) | 0.91  (0.78-1.04) |
|  | **Mean distance (km), trips by private car (95% UI)** | 15.94  (15.35-16.36) | 13.89  (12.25-15.75) | 14.01  (12.98-15.17) | 11.25  (10.15-12.50) | 11.05  (8.71-13.60) | 9.57  (7.40-12.16) |
| **Females** | **Proportion of population, baseline** | 61% | 4% | 16% | 13% | 4% | 2% |
|  | **Proportion of population, Scenario 1** | 59% | 3% | 14% | 13% | 7% | 4% |
|  | **Proportion of population, Scenario 2** | 54% | 1% | 4% | 11% | 11% | 19% |
|  | **Mean number PT trips taken per day**  **(95% UI)** | 0.16  (0.15-0.18) | 0.23  (0.18-0.29) | 0.27  (0.24-0.31) | 0.41  (0.37-0.45) | 0.45  (0.39-0.53) | 0.66  (0.50-0.92) |
|  | **Mean distance walked (km), for combined PT access/egress (95% UI)** | 1.19  (1.11-1.29) | 1.08  (0.94-1.24) | 1.10  (1.02-1.19) | 1.09  (1.03-1.17) | 1.11  (1.01-1.26) | 1.26  (1.11-1.43) |
|  | **Mean distance (km), trips by private car (95% UI)** | 12.35  (11.95-12.76) | 10.85  (9.54-12.42) | 10.06  (9.40-10.80) | 9.62  (8.47-10.82) | 8.71  (7.01-10.60) | 8.08  (6.15-10.42) |

*Tables notes:* km=kilometres. PT=public transport. 95% UI=95% uncertainty interval.
