# Supplementary material for: Better transport accessibility, better health: a health economic impact assessment study for Melbourne, Australia
Source: Int J Behav Nutr Phys Act. 2019 Oct 22;16:89. doi: 10.1186/s12966-019-0853-y (PMC6805526; doi:10.1186/s12966-019-0853-y)
Supplement: Supplementary file 3 — Additional file 3. Estimation of physical activity and obesity effect. Provides estimates of physical activity and obesity effect. (DOCX 17 kb) [file 12966_2019_853_MOESM3_ESM.docx]

**Additional file 3 – Estimation of physical activity and obesity effect**

Table S3.1 – Intervention effect per head of population, modelled to physical activity and body mass index effect

| **Scenario** | **Age (years)** | **Time spent to access PT per week (mins), per capita** | **METs to kcal/min, per capita** | **kJ per week from intervention, per capita** | **Weight effect (kg), per capita** |
| --- | --- | --- | --- | --- | --- |
| **Males** | | | | | |
| **Scenario 1** | **20-24** | 1.55 | 2.07 | 13.46 | -0.02 |
|  | **25-29** | 2.78 | 2.20 | 25.63 | -0.04 |
|  | **30-34** | 1.47 | 2.20 | 13.58 | -0.02 |
|  | **35-39** | 1.30 | 2.31 | 12.59 | -0.02 |
|  | **40-44** | 0.34 | 2.35 | 3.35 | -0.005 |
|  | **45-49** | 1.10 | 2.29 | 10.54 | -0.02 |
|  | **50-54** | 0.24 | 2.30 | 2.32 | -0.003 |
|  | **55-59** | 1.07 | 2.31 | 10.30 | -0.01 |
|  | **60-64** | 0.21 | 2.30 | 2.06 | -0.003 |
|  | **65-69** | 1.38 | 2.27 | 13.11 | -002 |
|  | **70-74** | 0.75 | 2.26 | 7.08 | -0.01 |
| **Scenario 2** | **20-24** | 10.32 | 2.07 | 89.46 | -0.13 |
|  | **25-29** | 16.38 | 2.20 | 150.78 | -0.22 |
|  | **30-34** | 4.04 | 2.20 | 37.22 | -0.05 |
|  | **35-39** | 3.67 | 2.31 | 35.46 | -0.05 |
|  | **40-44** | 1.09 | 2.35 | 10.76 | -0.02 |
|  | **45-49** | 6.85 | 2.29 | 65.58 | -0.09 |
|  | **50-54** | 1.95 | 2.30 | 18.80 | -0.03 |
|  | **55-59** | 2.54 | 2.31 | 24.56 | -0.04 |
|  | **60-64** | 2.15 | 2.30 | 20.70 | -0.03 |
|  | **65-69** | 5.12 | 2.27 | 48.67 | -0.07 |
|  | **70-74** | 6.36 | 2.26 | 60.09 | -0.09 |
| **Females** | | | | | |
| **Scenario 1** | **20-24** | 0.99 | 1.77 | 7.33 | -0.01 |
|  | **25-29** | 0.97 | 1.83 | 7.39 | -0.01 |
|  | **30-34** | 1.57 | 1.83 | 11.99 | -0.02 |
|  | **35-39** | 0.58 | 1.86 | 4.56 | -0.01 |
|  | **40-44** | 0.35 | 1.81 | 2.79 | -0.004 |
|  | **45-49** | 0.62 | 1.96 | 5.06 | -0.01 |
|  | **50-54** | 0.34 | 1.88 | 2.69 | -0004 |
|  | **55-59** | 0.48 | 1.91 | 3.84 | -0.01 |
|  | **60-64** | 0.59 | 1.95 | 4.84 | -0.01 |
|  | **65-69** | 0.48 | 1.93 | 3.84 | -0.01 |
|  | **70-74** | 0.63 | 1.92 | 5.08 | -0.01 |
| **Scenario 2** | **20-24** | 7.56 | 1.77 | 56.09 | -0.08 |
|  | **25-29** | 7.36 | 1.83 | 56.41 | -0.08 |
|  | **30-34** | 7.66 | 1.83 | 58.59 | -0.08 |
|  | **35-39** | 3.30 | 1.86 | 25.75 | -0.04 |
|  | **40-44** | 2.11 | 1.81 | 16.87 | -0.02 |
|  | **45-49** | 3.21 | 1.96 | 26.28 | -0.04 |
|  | **50-54** | 3.21 | 1.88 | 25.23 | -0.04 |
|  | **55-59** | 3.41 | 1.91 | 27.19 | -0.04 |
|  | **60-64** | 3.60 | 1.95 | 29.32 | -0.04 |
|  | **65-69** | 4.97 | 1.93 | 40.03 | -006 |
|  | **70-74** | 8.04 | 1.92 | 64.69 | -0.09 |

Table notes: kcal=kilocalories. kg=kilograms. kJ=kilojoules. M=metres. METs=metabolic equivalent tasks. PT=public transport.
